# A qualitative study of same session co-use of nicotine and cannabis among adolescents and young adults

**DOI:** 10.1371/journal.pone.0340050

**Published:** 2026-01-07

**Authors:** Danielle R. Davis, Dana A. Cavallo, Krysten W. Bold, Meghan E. Morean, Grace Kong, Wei Li, Vanessa Ponte, Nicholas Franco, Sarah Lichenstein, Suchitra Krishnan-Sarin

**Affiliations:** Department of Psychiatry, Yale School of Medicine, New Haven, Connecticut, United States of America; University of Wisconsin-Madison, UNITED STATES OF AMERICA

## Abstract

**Purpose:**

Nicotine and cannabis are commonly co-used among adolescents and young adults (AYAs). Same session co-use has more negative effects than co-use that does not overlap. Little is known about how or why these products are used within the same session. Enhanced understanding of this behavior pattern can be leveraged to reduce co-use in this population.

**Methods:**

Connecticut AYAs (15–20 years old) who self-reported past-month nicotine and cannabis vaping participated in 1-hour focus groups examining of nicotine and cannabis use. Six focus groups were conducted in Spring 2023 (N = 29, mean group size n = 5). Data was analyzed with a two-stage deductive and inductive approach.

**Results:**

Participants reported deliberate same session use in various modes (e.g., smoking, vaping). Same session use was most commonly reported to enhance positive psychoactive effects of cannabis (e.g., improve/enhance the high). Other common reasons were to reduce negative cannabis effects, such as vaping nicotine to reduce throat irritation or mask taste (e.g., vaping flavored nicotine product to mask cannabis taste). Some reported vaping nicotine so frequently that it unintentionally overlapped with cannabis use. Finally, other participants reported avoiding same session use as the combined psychoactive effects of nicotine and cannabis were too strong.

**Conclusion:**

AYAs reported same session use primarily to enhance cannabis use experience (e.g., improving high or taste, reducing negative effects). Our findings indicate a complementary role of nicotine and cannabis, and suggest that prevention and cessation efforts for either substance need to address co-use behaviors.

## Introduction

Nicotine and cannabis are the most-used substances among adolescents and young adults (AYAs) [1,2], and co-use (i.e., current use of both nicotine and cannabis) is well-documented in this population [3–6]. Use of nicotine and cannabis are independently associated with negative physical and psychosocial outcomes (e.g., dependence, reduced cognitive function, lung problems) [7–11]. Among AYAs, vaping is the most common form of nicotine administration [12–15] and data from 2023 demonstrate that 10.0% of high school students report past-month nicotine vaping [16]. Among that group, over one-third report frequent vaping (i.e., ≥ 20 days out of the past 30 days) [16], which is of concern given the link between nicotine use frequency and dependence among this population [17] and the potential harms of nicotine vaping among young people including quicker acquisition of nicotine dependence, behavioral problems (e.g., increased impulsivity), cardiovascular symptoms, and increased adverse respiratory symptoms [7,18].

Cannabis use prevalence is also high among AYAs with rates of past-month use reported at 20.2%, 12.1%, and 5.0% among 12^th^, 10^th^, and 8^th^ graders in 2022, respectively [1]. Routes of cannabis administration can include smoking (e.g., via blunts, joints, spliffs), vaping, dabbing (e.g., use of a dab rig to inhale concentrated cannabis extracts), edible use, and topical use. With the emergence of vaping cannabis over the last decade, cannabis routes of administration among AYAs appears to have shifted. While smoked cannabis has historically been the most common route of consumption, it appears to be decreasing in prevalence over time with vaping cannabis increasing in prevalence over time among this population [19]. Among AYAs who vape nicotine, cannabis vaping appears especially prevalent; with over half of high school-aged youth who vape nicotine also reporting cannabis vaping [20–23]. However, there are data suggesting that AYAs who vape nicotine also use other methods of cannabis administration (e.g., smoking, edible) [4].

“Co-use” of nicotine and cannabis is a broad term that can refer to a wide range of behavioral patterns [24], including current use of both products on different days (e.g., cannabis use on one day, nicotine use on another), use on the same day at different times (e.g., nicotine use in the morning, cannabis use in the evening), and use on the same day within the same session (e.g., use of both cannabis and nicotine within the same time period). Same session nicotine and cannabis use is an important pattern of behavior to investigate given that substances may interact during same session use to produce unique effects. Emerging data from AYAs indicate that same session use may be to elicit enhanced psychoactive effects [5,25]. Importantly same session use can be comprised of multiple types of co-use behaviors, as previously noted in qualitative work with young adults [25]. These behaviors include same occasion sequential co-use (e.g., vaping nicotine followed by use of a cannabis product) or same occasion simultaneous use (e.g., use of a blunt which contains tobacco in the wrapper and cannabis filled within the wrapper) [25].

While same session nicotine and cannabis use is observed among AYAs who vape nicotine, there is still a need to focus on the spectrum motives behind these behaviors and use patterns to characterize same session use. Additionally, given that nicotine vaping is the most common form of nicotine use among AYAs and cannabis use is also common in this population, there is a need to understand how nicotine vaping may influence patterns of cannabis co-use. Additionally, as vaping cannabis continues to grow in popularity among AYAs who vape nicotine, it is important to understand how the rise of this route of administration might differ from previously established co-use behaviors. Past qualitative data on nicotine and cannabis co-use prior to the rise of vaping of either substance indicate same session use to be simultaneous and associated with positive perceptions of cannabis and negative perceptions of nicotine, as well as use of nicotine products as a delivery method of cannabis (e.g., blunts) [26], but as other research has indicated vaping as a route of administration may shift co-use behaviors (e.g., the rise of sequential use with vaped products) [5,25] and may impact product perceptions.

Understanding this relationship can advance interventions for both nicotine and cannabis use and inform regulations on both substances (e.g., impact of restrictions of one product on use of the other). There is also a gap in understanding how intentions to use each substance influence same session use, if there are additional motivations to engage in same session use beyond those previously identified (e.g., psychoactive effects [5,25]), and how product type (e.g., vaping, smoking) influences same session use. Thus, the current study utilized focus group data from AYAs who report current nicotine and cannabis vaping to explore motivations and patterns of same session use.

## Materials and methods

### Participants

We aimed to recruit high school students (ages 13–18) and young adults (18–20) who reported past-month nicotine vaping and past-month cannabis vaping and recruited from March 03 2023 to July 10 2023. High school students were recruited in-person at local Connecticut high schools. We recruited across a range of suburban and urban school districts that fell in the bottom third of Connecticut’s District Reference Groups (DRG), a classification system that groups Connecticut schools by socioeconomic status (SES). Schools in the bottom third DRGs have a lower SES and a higher proportion of Black, Indigenous, People of Color (BIPOC) students [27]. Young adults who had completed high school were recruited from the community via a database of people who had previously screened for studies within our group and were interested in future studies. For underage minors (ages <18), passive parental permission was obtained through emails distributed by the schools. Parents were sent information about studies conducted by our group and were given the option to opt their student out of participating in these studies. The Yale Institutional Review Board approved this study (Human Investigations Committee Number: 207010580).

### Screening questionnaire

A brief online screener was used to obtain demographic information (age, high school enrollment status, gender, race, ethnicity) and lifetime and past-month use of nicotine e-cigarettes, cannabis products, and other tobacco products/substances (i.e., alcohol, cigarettes, cigars/cigarillos/little cigars, hookah, smokeless tobacco, and nicotine pouches). Lifetime and past-month use of the following routes of cannabis administration were assessed: vaping flower/bud, vaping concentrates/dab pens, dabbing (via traditional dab rig), smoking cannabis, edible use, tinctures, topical use, or consuming raw/juiced cannabis. For frequency of use, number of days of use in the past 30 days was collected for all participants.

### Focus group procedures

Focus groups were split into high school students (n = 3) and young adults not enrolled in high school (n = 3). The 1-hour focus groups were conducted virtually via HIPAA secure Zoom from March-July 2023. Author DC is a clinical psychologist with 20 years of experience conducting youth and young adult focus groups [28–31]. Author DRD co-facilitated 5 of the groups and author SL co-facilitated 1 of the groups. Both are PhD level psychologists, have experience with qualitative data collection, and received training and instruction by author DC prior to co-facilitation.

At the beginning of each focus group, verbal assent (<18 years old) or consent (≥18 years old) was obtained. Participants were informed that groups would be audio recorded, were instructed to keep information shared in the groups private, and were informed they could stop participation at any time. Participants were compensated with a $50 Amazon e-gift card. All procedures were approved by the Yale Human Investigations Committee (HIC # 12070010580).

A focus group guide (**File in** S1 File) was developed a priori to assess nicotine and cannabis product use, and the structure of the guide was informed by a prior focus group guide assessing patterns of youth nicotine e-cigarette use [29,32]. The guide covered several content areas including same session nicotine and cannabis use utilizing content assessing how nicotine and cannabis were used within the same session, what was like/disliked about same session use, and reasons for same session use. While nicotine and cannabis vaping were the primary areas of interest of the groups, AYAs were encouraged to report all types of nicotine and cannabis use.

### Data analyses

Zoom audio recordings were transcribed using Rev, a transcription service. Transcriptions were manually reviewed by the research team to correct any errors. Data analysis was conducted using NVivo. We used the two-stage deductive and inductive approach, consistent with prior focus group research ^e.g.,^ [29,32]. Transcripts were analyzed thematically. The deductive approach required obtaining major themes *a priori*, which were first identified using the focus group guide and included type of same session use, reasons for same session use, and reasons for disliking same session use. Then, an inductive approach was used to identify any new themes by reviewing the transcripts. Transcripts were reviewed by the lead author (DRD) and coding team (Authors VP, NF) to identify additional themes and codes. First, VP and NF both independently coded a focus group that was determined to be the richest using the pre-specified codebook. New themes and codes that emerged, as well as uncertainty about whether identified text fit into certain themes or codes or coding discrepancies, were amended via group discussion with coding team. Once coding was consistent across coders, each independently coded the remaining transcripts. After coding was complete, the most common themes across focus groups were identified. We did not separate analyses by age group as no themes emerged that were unique for a particular age group.

## Results

### Participant characteristics

We completed six focus groups (N = 29; Group_M _= 5 (SD:1.7)). Participants were on average 17.9 (SD: 1.7) years old and 41.4% were in high school. 55.2% reported gender as man/boy, 41.4% as woman/girl, 3.4% as non-binary, and 3.4% as transgender. Participants identified as White (75.9%), Black (13.8%), Asian (6.9%), Pacific Islander/Native Hawaiian (3.4%), from another race (17.2%), and could endorse more than one race Additionally, 24.1% of participants reported Hispanic ethnicity. All participants reported past-month nicotine vaping, with an average of 18.9 (SD: 11.6) days of use. All but one participant reported past-month cannabis use; this individual reported past-year use. The average number of days of use in the past month was 16.7 (SD: 10.3). For cannabis, the most common type of past-month use was use of vaped cannabis concentrates/dab (86.2%), followed by cannabis smoking (79.3%). Full demographic and product use characteristics are reported in **Table 1**.

**Table 1:** Substance use characteristics of focus group participants (n = 29).

Product Use	Lifetime (%)	Past-Month (%)	Past-Month Days of Use, M (SD)
Nicotine Vaping	100.0%	100.0%	18.9 (11.6)
Any Cannabis Use	100.0%	96.6%	16.7 (10.3)
Modes of Cannabis Use^a^			
Vaping Concentrates/Dab Pen	96.6%	86.2%	13.8 (10.9)
Smoking	93.1%	79.3%	10.9 (9.4)
Edible	86.2%	31.0%	2.6 (6.5)
Vaping Flower/Bud	62.1%	34.5%	9.5 (12.1)
Dabbing	55.2%	24.1%	4.4 (6.0)
Raw Juiced	13.8%	3.4%	2.5 (5.0)
Tincture	20.7%	0.0%	---
Topicals	13.8%	0.0%	---
Non-Cannabis Product Use			
Alcohol	93.1%	72.4%	8.4 (5.3)
Cigarettes	62.1%	17.2%	4.0 (3.5)
Hookah	37.9%	10.3%	4.0 (3.0)
Nicotine Pouch	34.5%	10.3%	11.0 (16.5)
Cigars	31.0%	3.4%	1.0 (0)
Smokeless Tobacco	10.3%	0.0%	

^a^Modes of cannabis delivery included the following definitions: vaping flower/bud, vaping concentrates (e.g., oil, wax) or using a dab pen), dabbing (with a traditional dab rig and torch/nail), smoking (blunt, joint, pipe, bong), eating “edibles” (brownies, candies, or other foods/beverages), using tinctures (liquid that is usually placed under the tongue), using topicals (lotions or balms), eating or drinking raw or juiced marijuana.

### Nicotine and cannabis product use

During the focus groups, participants primarily described vaping nicotine using disposable vaping devices like Elf Bar and Crave, which typically advertise several thousand puffs of product, come in sweet and non-tobacco flavors, and are at a relatively low price point (e.g., $10-$15). Although not common, use of other tobacco products was mentioned including oral nicotine pouches (e.g., white discreet nicotine-filled pouches that are placed between the gum and the lip, Zyn being a brand mentioned in the current focus groups) and cigarettes (**Table 1**).

Regarding cannabis use, both vaping and smoking were commonly discussed. Cannabis vaping devices were often referred to as “carts” – a shortening of the word “cartridge” – to reference the liquid-filled cartridge in cannabis vaping devices. Participants described a variety of methods of smoking cannabis, including via bowls, bongs, and joints/spliffs. Infrequently, participants discussed using dab rigs, in which a cannabis concentrate is added directly to a heating element. Notably, products containing both nicotine and cannabis were described, including blunts (i.e., cannabis flower or a cannabis-tobacco mixture filled in hollowed out cigars), and “chop(s)” (i.e., mixing cigarette/loose leaf tobacco with cannabis for use in a bong).

### Same session use

Same session use was endorsed across all six focus groups. Two types of same session use that reflected intentions to engage in same session use were identified: intentional and incidental same session nicotine and cannabis use. Intentional same session use was defined as same session use with a distinct motivation or reason. Incidental same session nicotine and cannabis use refers to same session use which is not planned or due to a specific reason or motive (See **Table 2** for example quotations). Much of the incidental same session use was due to vaping nicotine so frequently or so habitually during the day that it overlapped with cannabis use at the same session without intentionally planning to do so. For example, one focus group participant stated “Like, it’s just that I vape [nicotine] so much in my everyday life, that being high [through cannabis use], it’s no different. I’m still gonna [sic] reach for my pen [nicotine], take a hit. It’s not, like, an intentional thing, where I’m doing them simultaneously; it’s more just, like, that’s just what I do when I’m not high.”. Both intentional and incidental use were discussed in the focus groups, but intentional same session use was more common than incidental same session use.

**Table 2:** Reasons for same session use.

Same Session Use Pattern	Reason/Motivation for Same Session Use	Exemplar Quotations
Incidental Same Session Use	---	“I feel like when I’m smoking [cannabis], when I go for my nicotine, I’m just gonna [sic] do that anyways. It’s not necessarily because I’m smoking [cannabis]” “When I do it in conjunction [meaning same session nicotine and cannabis use], I’m not necessarily doing it in conjunction on purpose. Like, it’s just that I vape [nicotine] so much in my everyday life, that being high, it’s no different. I’m still gonna [sic] reach for my pen [nicotine], take a hit. It’s not, like, an intentional thing, where I’m doing them simultaneously; it’s more just, like, that’s just what I do when I’m not high.”
Intentional Same Session Use		
	Psychoactive Effects	“I’ll be, like, ripping a [cannabis] pen and, like, okay, now I’m high. And then, like, the, the nicotine will kind of like intensify the effects or, like, change it in a little way, like a more positive way…like, more pleasurable effects, so that, like, it’s like weed is the base and the nicotine is kind of built on top of that, if that makes sense.” “I’ll hit my [cannabis] pen and then hit nicotine ‘cause it’s an enhancer. It boosts it, so, like, if you’re not feeling the marijuana as much as, like, you want to, you hit the nicotine a little bit.” “Including nicotine in with the weed. I don’t know how to explain it. It just produces a like, a nice, calming feeling because weed already relaxes you and so the nicotine relaxes you, as well, but also adds a buzz that I think weed doesn’t really have.”
	Flavor	“Nicotine is a chaser for weed. So, like, if you’re hitting, like, your friend’s cart and it’s just some sort of awful, like, low-level strain and it tastes so bad, you can just taste blue raspberry afterwards and it’s a lot better.” “I mix it [nicotine] in most of the times to like get a better flavor or like get the wood out of my taste, like get the wood taste out.”
	Reducing Aversive Physical Effects	“Like if I’m smoking weed, I am more- like 100% more likely to hit my [nicotine] vape like immediately after, because it soothes my throat. Like especially if it’s something like menthol or cool mint, like it just gives me a better- a better feeling in my throat.” “When I like take a long [cannabis] cart rip, I usually like use my nic [sic] right after so I don’t cough.”
	Social Use	“In party settings, you can kind of see the people, like, who’s gonna do the most daring thing, and they’ll mix, like, whatever.”
	Access	“I’ve been to parties and it’s just a frequent thing there. People will just be like, passing around whatever they have, and just, like, in, like, a complete, like, rotation, just like, taking whatever’s in front of them.”

#### Reasons for intentional same session use.

Reasons for intentional same session use were coded into the following categories: Psychoactive Effects, Flavor, Reducing Aversive Physical Effects, Social Use, and Access (See **Table 2** for example quotations). Psychoactive Effects was the most commonly described reason for intentional same session use, with most descriptions related to using nicotine following cannabis to enhance or improve the cannabis high. For example, one focus group participant noted: “The nicotine will kind of like intensify the [cannabis] effects or, like, change it in a little way, like a more positive way…like, more pleasurable effects, so that, like, it’s like weed is the base and the nicotine is kind of built on top of that, if that makes sense.” Nicotine was described as ‘enhancing’, ‘heightening’, ‘evening out’, or ‘complementing’ the experience of cannabis use by adding a pleasant nicotine ‘buzz’ to the cannabis high. Other ways this was described was as providing a ‘stronger’ psychoactive effect of cannabis when nicotine was added. Altered psychoactive effects were typically reported with sequential use (e.g., use of a nicotine vape following cannabis administration across both smoking and vaping), but there was also some report of heightened psychoactive effects when products were smoked simultaneously and specifically there was a report of a ‘faster’ onset of psychoactive effects when cannabis and nicotine were used simultaneously in a blunt. Less commonly was discussion of that nicotine and cannabis in the same session enhanced nicotine’s effects including the “nicotine buzz.” Specifically, this was noted as nicotine buzz being less distinguishable without cannabis and cannabis use “bringing back the buzz of nicotine.”

Flavor was another reason for engaging in same session use. Participants unanimously described using nicotine during or following cannabis use to remove or mask the aversive taste of cannabis. For example, some participants reported using a nicotine e-cigarette device with a flavor (e.g., blue raspberry) following cannabis administration to mask the taste (e.g., as a “chaser”), while other descriptions were of combustible tobacco mixed with cannabis to mask the “woody” flavor of cannabis. Notably, the pattern of sequential same session use of a flavored nicotine vape following the use of cannabis was described to remove the aversive taste of both vaped and smoked cannabis.

Similarly, reasons for same session use that were related to reducing the Aversive Physical Effects of Cannabis included using vaped nicotine to reduce/mask the aversive physical effects of cannabis in the mouth and throat, including irritation and the urge to cough. For example, one participant reported that they used menthol/mint flavored nicotine e-cigarettes to soothe their throat after cannabis use, another reported using their nicotine vape to reduce the experience of dry mouth that accompanies using smoked cannabis, and others still reported that using the nicotine vape in between cannabis uses enhances the smoothness of the experience and states: “I do have friends.. that’ll, like, smoke weed and kind of like in between hits…, they will kind of take a hit… a [nicotine] vape and they’ll claim that… it kind of helps with the throat feel. It helps, like, go down smoother.”

Uncommonly discussed reasons for same session use included Social Use and Product Access, which overlapped in described content. Same session use occurred in party settings where friends/peers were sharing blunts and/or vaped nicotine and cannabis. This was described as products being shared and both simultaneous (e.g., blunt) and sequential same session use occurred in social settings this way. Other social use was described as people trying to show off to peers by mixing nicotine and cannabis or using products they may not otherwise use in the same session with a participant describing: “Who’s gonna [sic] do the most daring thing, and they’ll mix…., I guess pride thing in that sense.” Access overlapped with social use as same session use was described as more likely to occur and thus be more accessible in social situations, namely parties (See **Table 2** for additional quotes).

#### Reasons to avoid same session use.

Reasons to avoid same session nicotine and cannabis use were also discussed. These themes included Negative Psychoactive Effects and Negative Physical Effects. Negative psychoactive effects were more commonly discussed. This was typically described as the combined effect being too intense or uncomfortable. Reasons for experiencing these negative effects included self-reported low tolerance to both substances making combining them aversive or in the participants words “send me over the edge”. Additionally, in contrast to those who reported using nicotine and cannabis in the same session to enhance cannabis high, others reported avoiding same session use because they felt it lessened the effects of cannabis. Participants described mixing cannabis and nicotine e-liquid in a vaping device which produced what they described as “a diluted form of weed” or using nicotine and cannabis together in a smoked form which was described as “very filtered and it didn’t feel like smoking weed to me.” Negative physical effects included lung pain associated with simultaneous same session use (i.e., use of ‘chops’)and nausea. Nausea was described as being amplified with a participant stating: “I feel like the nausea associated with nicotine is really amplified when you are high [from cannabis use]”.

## Discussion

This study used focus group data to identify patterns of and reasons for same session nicotine and cannabis use among AYA who vaped nicotine. Same session use was commonly reported in the focus groups, with distinct patterns emerging; for example, some experiences involved intentionally using both products within a session and other instances of incidentally using both products in the same session. These patterns have important implications for public education and intervention development as there may be different subgroups who benefit from targeting cannabis and nicotine as complements (i.e., the use of one substance increases use of the other) and/or as independent substances (i.e., use of one does not impact use of the other). During discussion of intentional use, participants directly note that cannabis and nicotine complement each other. Importantly, while both nicotine and cannabis were reported as being used for the purpose of complementing the other substance, there was a common theme of nicotine being used as a complement to cannabis versus cannabis being used to complement nicotine.

Regarding reasons for intentional same session use, augmented psychoactive effects were commonly described. Increased psychoactive effects from same session use have been previously observed in other qualitative work among adolescents [5] and young adults [25]. In our study, this was also observed with participants primarily discussed using nicotine to enhance the cannabis ‘high’ or to increase pleasurable effects of cannabis. Notably, this sample did not report needing to use nicotine to make cannabis use pleasurable, but rather as a way to enhance the positive experience. It is unclear if cannabis use would decrease among this group if nicotine was less accessible. It seems likely they would still use cannabis, but targeting nicotine, which in turn may make cannabis less pleasurable may be one method to make cannabis easier to quit.

While some participants reported using nicotine to enhance the positive effects of cannabis, others indicated a different experience with same session use. For reasons to avoid same session use, psychoactive effects were also noted, with some participants explaining that co-use made the effects of cannabis too strong while others indicated that co-use made the effects of cannabis too weak. This is notable because these reasons for avoiding same session use are discrepant from each other and may reflect characteristics of the user (e.g., experience with the nicotine and/or cannabis, regularity of use of nicotine and/or cannabis) or the product itself (e.g., potency of the cannabis). For example, in the groups, one report of same session use being aversive because of strong psychoactive effects came from a participant who self-reported a “low tolerance”, which may indicate characteristics of the user, while another reported aversive effects due to the low potency of the cannabis when mixed with nicotine indicating perceived characteristics of the product itself (**Table 3**). Importantly, recruitment was restricted to those with a history of cannabis vaping given the focus of the broader study on identifying people who vape both nicotine and cannabis. However, we observed that this dual-use population also reported a wide range of cannabis administration routes, prompting us to include broader cannabis administration patterns [4]. Given participants’ familiarity with both nicotine vaping and cannabis products, frequent use of both substances, and likely dependence to both substances, it is possible that that we did not capture the full spectrum of aversive or negative experiences with same session use. It will be important to investigate if same session use is more common in AYAs with higher dependence on either substance and how dependence and frequency of use impacts same session use behaviors.

**Table 3:** Reasons to avoid same session use.

Negative Psychoactive Effects	“I have, like, a low tolerance in terms of, like, any substance, so yeah, if I’m smoking weed and I already feel high, like, I’m not gonna even try to hit... the disposable nicotine because I know it’s gonna, like, send me over the edge” “It’s like a major head rush that like could be seen as a good thing, but sometimes, like, it’s too much” “The weed wasn’t as strong because it was very filtered with tobacco and it just didn’t feel like smoking weed to me”
Negative Physical Effects	“Taking a chop, it hurts your lungs a little bit.” “A Lotta [sic] people often get nauseous, because I feel like the nausea associated with nicotine is really amplified when you’re high.”

Importantly, intentional same session use was also reported to negate aversive experiences associated with cannabis use, including negative physical effects and unpleasant taste. Prior research with young adults indicates that nicotine is used to reduce an unpleasantly strong cannabis high [25]. However, in our study, participants described nicotine’s use to reduce aversive cannabis effects as mostly related to sensory experiences in their mouth and throat (e.g., coughing, throat irritation, aversive flavor). There was discussion of using flavored nicotine e-cigarettes following cannabis smoking to combat the aversive taste of cannabis, which is a novel finding in this study. Cooling flavors in nicotine e-cigarettes, including menthol and mint, were noted as particularly relevant to reduce cannabis-related irritation in the mouth and throat. Menthol and mint flavors are well-known to reduce nicotine related harshness and irritation in tobacco products [33–36], and this study is the first to demonstrate that these constituents are being used for a similar reason across substances. Additional research is needed to inform how the restriction of flavored nicotine e-cigarettes could impact this co-use behavior. This study was conducted in Connecticut, where flavors in nicotine e-cigarettes are not restricted, and both sweet and cooling flavors are available. It may be useful to examine if this pattern of behavior persists in places where e-cigarette flavors are restricted (e.g., Massachusetts, California) or if AYAs in these locations engage in other behaviors to reduce these aversive effects of cannabis. It was noted by one participant that nicotine vaping in general made the same session use ‘smoother’ without specifying or noting a particular flavor that produced smoothness, so it may be that for some flavor is less relevant although this is somewhat unclear. Additionally, use of these products for reduction of aversive taste or physical effect produces a unique pattern of behavior with repeated sequential use within the same session. Participants would report taking nicotine immediately after cannabis uses repeatedly, which indicates that sequential use may occur several times within a single session.

This study includes some limitations. As this is a qualitative study, additional quantitative analyses would help to examine how commonly AYAs endorse these reasons of same session use, as well as identify characteristics predictive of same session use. Additionally, AYAs were recruited from Connecticut where cannabis is legal for adult use (i.e., 21+) and no flavor or nicotine concentration restrictions exist for nicotine e-cigarettes, which may limit generalizability for other jurisdictions in which cannabis is not recreationally legal and/or have e-cigarette restrictions. However, it should be noted that many of the young adult sample who participated in our study either only resided in CT to attend college or were from CT but attended college out of state, thus their use experience may be more broadly representative. While the target population was current (past-month) users of cannabis and nicotine, we did include a participant with past-year, but not current cannabis use to complete the final focus group. Due to the nature of the data collection, we could not conduct a sensitivity analyses to assess their individual impact on the results. Finally, recruitment was restricted to those with a history of cannabis vaping, which may have somewhat limited the generalizability of the sample.

In sum, same session cannabis use was observed among AYAs who vaped nicotine and used cannabis. Cannabis and nicotine co-use is well established to be associated with a range of negative user effects and potential negative long-term outcomes including increased risk of cannabis and/or tobacco use disorder and same session use may uniquely impact risk of adverse outcomes [3,37]. Given that access to cannabis is increasing due to the changing legal landscape of cannabis in the U.S., it is important to monitor AYAs who engage in nicotine vaping to understand how their nicotine use may impact their cannabis use behaviors and vice versa. Additionally, when considering interventions or regulations aimed at either substance, understanding co-use patterns can inform the development and targeting of interventions, as well as impact regulations targeting nicotine or cannabis products.

## Supporting information

S1 File Focus group guide created by authors in Winter-Spring 2023. (DOCX)

## References

[1] Johnston LD, Miech RA, Patrick ME, O’Malley PM, Schulenberg JE, Bachman JG. Monitoring the Future National Survey Results on Drug Use, 1975-2022: Overview, Key Findings on Adolescent Drug Use. Institute for Social Research. 2023.

[2] Patrick ME, Miech RA, Johnston LD, O’Malley PM. Monitoring the Future Panel Study Annual Report: National Data on Substance Use among Adults Ages 19 to 60, 1976-2022. Institute for Social Research. 2023.

[3] Yimer TM, McClure-Thomas C, Stjepanovic D, Wilson J, Chan GCK, Hall WD, et al. The relationship between cannabis and nicotine use: A systematic review and meta-analysis. Addiction. 2024;119(12):2076–87. doi: 10.1111/add.16642 39129583

[4] Davis DR, Bold KW, Wu R, Morean ME, Kong G, Krishnan-Sarin S. Use of cannabis among youth who vape nicotine. Addict Behav. 2025;160:108173. doi: 10.1016/j.addbeh.2024.108173 39326231 PMC12970032

[5] Davis DR, Bold KW, Kong G, Cavallo DA, Jackson A, Krishnan-Sarin S. Cannabis use among youth who vape nicotine E-cigarettes: A qualitative analysis. Drug Alcohol Depend. 2022;234:109413. doi: 10.1016/j.drugalcdep.2022.109413 35339972 PMC9018562

[6] Reboussin BA, Wagoner KG, Ross JC, Suerken CK, Sutfin EL. Tobacco and marijuana co-use in a cohort of young adults: Patterns, correlates and reasons for co-use. Drug Alcohol Depend. 2021;227:109000. doi: 10.1016/j.drugalcdep.2021.109000 34507062 PMC8516030

[7] McGrath-Morrow SA, Gorzkowski J, Groner JA, Rule AM, Wilson K, Tanski SE, et al. The Effects of Nicotine on Development. Pediatrics. 2020;145(3):e20191346. doi: 10.1542/peds.2019-1346 32047098 PMC7049940

[8] Morean ME, Davis DR, Kong G, Bold KW, Talley A, Krishnan-Sarin S. Psychometric evaluation of the Self-Report Habit Index for assessing habitual e-cigarette use behavior in high school adolescents. Drug Alcohol Depend Rep. 2024;12:100251. doi: 10.1016/j.dadr.2024.100251 39050698 PMC11267024

[9] Morean ME, Krishnan-Sarin S, S O’Malley S. Assessing nicotine dependence in adolescent E-cigarette users: The 4-item Patient-Reported Outcomes Measurement Information System (PROMIS) Nicotine Dependence Item Bank for electronic cigarettes. Drug Alcohol Depend. 2018;188:60–3. doi: 10.1016/j.drugalcdep.2018.03.029 29753155 PMC6983293

[10] Chadi N, Minato C, Stanwick R. Cannabis vaping: Understanding the health risks of a rapidly emerging trend. Paediatr Child Health. 2020;25(Suppl 1):S16–20. doi: 10.1093/pch/pxaa016 33390752 PMC7757764

[11] Hall W, Leung J, Lynskey M. The Effects of Cannabis Use on the Development of Adolescents and Young Adults. Annu Rev Dev Psychol. 2020;2(1):461–83. doi: 10.1146/annurev-devpsych-040320-084904

[12] Delnevo C, Giovenco DP, Hrywna M. Rapid proliferation of illegal pod-mod disposable e-cigarettes. Tob Control. 2020;29(e1):e150–1. doi: 10.1136/tobaccocontrol-2019-055485 32001606 PMC7390658

[13] Gaiha SM, Lempert LK, McKelvey K, Halpern-Felsher B. E-cigarette devices, brands, and flavors attract youth: Informing FDA’s policies and priorities to close critical gaps. Addict Behav. 2022;126:107179. doi: 10.1016/j.addbeh.2021.107179 34861522 PMC8712419

[14] Gentzke AS, Wang TW, Jamal A, Park-Lee E, Ren C, Cullen KA, et al. Tobacco Product Use Among Middle and High School Students - United States, 2020. MMWR Morb Mortal Wkly Rep. 2020;69(50):1881–8. doi: 10.15585/mmwr.mm6950a1 33332300 PMC7745956

[15] Winickoff JP, Evins AE, Levy S. Vaping in Youth. JAMA. 2024;332(9):749–50. doi: 10.1001/jama.2024.13403 39110570

[16] Birdsey J, Cornelius M, Jamal A, Park-Lee E, Cooper MR, Wang J, et al. Tobacco Product Use Among U.S. Middle and High School Students - National Youth Tobacco Survey, 2023. MMWR Morb Mortal Wkly Rep. 2023;72(44):1173–82. doi: 10.15585/mmwr.mm7244a1 37917558 PMC10629751

[17] Vogel EA, Cho J, McConnell RS, Barrington-Trimis JL, Leventhal AM. Prevalence of Electronic Cigarette Dependence Among Youth and Its Association With Future Use. JAMA Netw Open. 2020;3(2):e1921513. doi: 10.1001/jamanetworkopen.2019.21513 32074292 PMC7780897

[18] Chaffee BW, Barrington-Trimis J, Liu F, Wu R, McConnell R, Krishnan-Sarin S, et al. E-cigarette use and adverse respiratory symptoms among adolescents and Young adults in the United States. Prev Med. 2021;153:106766. doi: 10.1016/j.ypmed.2021.106766 34418439 PMC8595821

[19] Wadsworth E, Craft S, Calder R, Hammond D. Prevalence and use of cannabis products and routes of administration among youth and young adults in Canada and the United States: A systematic review. Addict Behav. 2022;129:107258. doi: 10.1016/j.addbeh.2022.107258 35124565

[20] Dai H. Self-reported Marijuana Use in Electronic Cigarettes Among US Youth, 2017 to 2018. JAMA. 2020;323(5):473–4. doi: 10.1001/jama.2019.19571 31848567 PMC6990743

[21] Lim CCW, Sun T, Leung J, Chung JYC, Gartner C, Connor J, et al. Prevalence of Adolescent Cannabis Vaping: A Systematic Review and Meta-analysis of US and Canadian Studies. JAMA Pediatr. 2022;176(1):42–51. doi: 10.1001/jamapediatrics.2021.4102 34694342 PMC8546627

[22] Harrell MB, Clendennen SL, Sumbe A, Case KR, Mantey DS, Swan S. Cannabis Vaping Among Youth and Young Adults: a Scoping Review. Curr Addict Rep. 2022;9(3):217–34. doi: 10.1007/s40429-022-00413-y 35573056 PMC9078633

[23] Sun R, Mendez D, Warner KE. Evaluation of Self-reported Cannabis Vaping Among US Youth and Young Adults Who Use e-Cigarettes. JAMA Pediatr. 2022;176(4):417–9. doi: 10.1001/jamapediatrics.2021.6102 35129610 PMC8822439

[24] Hindocha C, McClure EA. Unknown population-level harms of cannabis and tobacco co-use: if you don’t measure it, you can’t manage it. Addiction. 2021;116(7):1622–30. doi: 10.1111/add.15290 33047862 PMC8041912

[25] Nguyen N, Islam S, Llanes KD, Koester KA, Ling PM. Classification of patterns of tobacco and cannabis co-use based on temporal proximity: A qualitative study among young adults. Addict Behav. 2024;152:107971. doi: 10.1016/j.addbeh.2024.107971 38281461 PMC10923078

[26] Akre C, Michaud P-A, Berchtold A, Suris J-C. Cannabis and tobacco use: where are the boundaries? A qualitative study on cannabis consumption modes among adolescents. Health Educ Res. 2010;25(1):74–82. doi: 10.1093/her/cyp027 19515745

[27] https://schoolstatefinance.org/drgs

[28] Cavallo DA, Kong G, Ells DM, Camenga DR, Morean ME, Krishnan-Sarin S. Youth generated prevention messages about electronic cigarettes. Health Educ Res. 2019;34(2):247–56. doi: 10.1093/her/cyz001 30753438 PMC6424147

[29] Kong G, Bold KW, Cavallo DA, Davis DR, Jackson A, Krishnan-Sarin S. Informing the development of adolescent e-cigarette cessation interventions: A qualitative study. Addict Behav. 2021;114:106720. doi: 10.1016/j.addbeh.2020.106720 33162230 PMC7785614

[30] Kong G, Bold KW, Simon P, Camenga DR, Cavallo DA, Krishnan-Sarin S. Reasons for Cigarillo Initiation and Cigarillo Manipulation Methods among Adolescents. Tob Regul Sci. 2017;3(2 Suppl 1):S48–58. doi: 10.18001/TRS.3.2(Suppl1).6 29085867 PMC5658780

[31] Kong G, Morean ME, Cavallo DA, Camenga DR, Krishnan-Sarin S. Reasons for Electronic Cigarette Experimentation and Discontinuation Among Adolescents and Young Adults. Nicotine Tob Res. 2015;17(7):847–54. doi: 10.1093/ntr/ntu257 25481917 PMC4674436

[32] Bold K, Kong G, Cavallo D, Davis D, Jackson A, Krishnan-Sarin S. School-based E-cigarette cessation programs: What do youth want?. Addict Behav. 2022;125:107167. doi: 10.1016/j.addbeh.2021.107167 34753093 PMC8629945

[33] Ahijevych K, Garrett BE. Menthol pharmacology and its potential impact on cigarette smoking behavior. Nicotine Tob Res. 2004;6 Suppl 1:S17-28. doi: 10.1080/14622200310001649469 14982706

[34] Kreslake JM, Wayne GF, Connolly GN. The menthol smoker: tobacco industry research on consumer sensory perception of menthol cigarettes and its role in smoking behavior. Nicotine Tob Res. 2008;10(4):705–15. doi: 10.1080/14622200801979134 18418792

[35] Krishnan-Sarin S, Green BG, Jordt SE, O’Malley SS. The Science of Flavor in Tobacco Products. 2019. PMC9896977 36743396

[36] Rosbrook K, Green BG. Sensory Effects of Menthol and Nicotine in an E-Cigarette. Nicotine Tob Res. 2016;18(7):1588–95. doi: 10.1093/ntr/ntw019 26783293 PMC4902888

[37] Tucker JS, Pedersen ER, Seelam R, Dunbar MS, Shih RA, D’Amico EJ. Types of cannabis and tobacco/nicotine co-use and associated outcomes in young adulthood. Psychol Addict Behav. 2019;33(4):401–11. doi: 10.1037/adb0000464 30985164 PMC6554032

