# Supplementary material for: A qualitative study of same session co-use of nicotine and cannabis among adolescents and young adults
Source: PLoS One. 2026 Jan 7;21(1):e0340050. doi: 10.1371/journal.pone.0340050 (PMC12779067; doi:10.1371/journal.pone.0340050)
Supplement: S1 File — (DOCX) [file pone.0340050.s001.docx]

**S1 File. Focus Group Guide**

**Focus Group Guide**

**Current Users**

**I. Introduction (5 Minutes)**

*Thank you all for joining this discussion to share your thoughts and opinions about marijuana vaping and nicotine vaping. We will talk about use of these products separately and then use of these products together. We are interested in learning about some of the reasons young people vape marijuana and nicotine. We want to eventually develop some program to help teens decrease their vaping.*

*Notes for Facilitators:*

- *Zoom room will open at 1:35 for participants; will tell them it will close promptly at 1:45; will lock room at 1:47*
- *Make sure to verbalize and emojis/nodding that is seen etc.*

*Notes for Participants:*

- *Participation in the group is 1 hr.*
- *Payment is $50 Amazon gift card. Treat this like a job etc.*
- *Please be in a quiet space where others are not around. Please try to stay in one space instead of moving around. Please silence your cell phones and avoiding texting/using your phone during the group (maybe note that we are doing the same).*
- *Cameras on & everyone should have JUST their first name showing. Danielle, co-facilitator, can help you change your name to just first name if needed*
- *The group chat is disabled, but you can chat hosts if need to.*
- *Feel free to use the raise hand function or other emojis if you agree with something someone else is saying.*
- *Try not to talk over each other and we want to hear from all of you.*
- *This Zoom will be audio recorded, but not video recorded. The recording will only be used to collect accurate data and will not be shared outside of our research group. The recording will be stored on a secure Yale server that only the research team has access to.*
- *While participating in the focus group, you will be asked to give your opinion about all types of vapes, including marijuana and nicotine vapes, as well as other marijuana products. I want to encourage everyone to be as honest and open as possible. Some of the information discussed in the focus group might be personal. Everything we discuss here is confidential, so please respect your peers and keep what is said in this room, in this room.*
- *There is no personal benefit for you to participate in the study. However, the information you provide may help with us to develop how we can help teens to quit marijuana and nicotine vaping.*
- *You are free to decide whether or not you want to participate in this focus group. If you do not want to participate, you are free to withdraw or leave the focus group at any time and it will not harm your relationship with your school or Yale University.*

*Does anyone have any questions before we start? Let’s start by going around the room and have everybody say their name and something about yourself.*

**II. Broad Discussion on Vaping (5 Minutes)**

- Tell me what people your age are vaping?
  - Marijuana, nicotine, or both?

**III. Discussion on Nicotine & Marijuana Vaping (20 Minutes)**

- Tell me about the vape products you use that contain nicotine.
  - Why/when do you vape nicotine?
  - What do you like/dislike about nicotine vaping?
  - What other tobacco products are you using, like cigarettes or cigars?
- Tell me about the vape products you use that contain marijuana.
  - What do you look for in a marijuana vape (e.g. type of marijuana, strength, other features)?
  - Why/when do you vape marijuana?
  - What do you like/dislike about marijuana?
    - Potential Likes:
      - it’s easy to hide than smoking
      - Flavor
      - The high
      - Calms you/reduces stress
      - Friends use it
      - Looks cool
      - Pain relief
      - Sleep help
      - Improving Mood
    - Potential Dislikes:
      - Cost
      - Loss of control
      - Adverse experience (i.e. bad high, anxiety)
      - Getting into trouble
  - What other marijuana products do you use besides vapes (e.g. bong, bowl, edibles etc.)?
    - What is your preferred way to use marijuana?
  - What other vape other products besides marijuana or nicotine (e.g. Delta-8, -10, CBD) do you use?
    - Why?

**IV. Discussion on co-use of Marijuana & Nicotine Vaping (15 Minutes)**

- *“Now we want to discuss similarities and differences of marijuana and nicotine vaping. We will also discuss if you ever use these products together. This includes times in which you used them one after the other (such as hit a nicotine vape pen and then used marijuana) or if you used them simultaneously (such as mixing vape products or putting tobacco in a joint etc.)”*
- How is vaping marijuana different from vaping nicotine? How are they similar?
  - What is your preference for either product and why?
- Tell us about your experience using marijuana and nicotine together.
  - When/why do you use them together?
  - What do you like about using them together? What do you dislike?
  - If no co-use, why do you not use them together?

**V. Discussion on Quit Attempts (15 minutes)**

- Have you ever wanted to quit or cut down vaping or using marijuana?
  - Have you tried to cut down or quit vaping?
  - Tell us about your experience.
    - How long they were able to quit/cut down
    - Products they quit/cut down (e.g. all marijuana products or something specific like vapes)
  - If cut/down quit, how did it make you feel? Did you have good or bad symptoms during this time?
    - Why did you resume marijuana use?
  - What did and didn’t help when you were quitting/cutting down?
  - Are you interested in quitting in the future?
- Do you think you could quit nicotine & marijuana vaping at the same time?
  - Why or why not?
- If no quit attempts:
  - What would make you want to quit?
  - What are some of the things you think you could gain from quitting?
  - If you were planning to quit vaping *marijuana or nicotine*, how would you go about doing it? What would be helpful?
    - Probe if they would quit all marijuana/nicotine products or just vaping and switch to other products

**VI. Discussion on Perceptions about Marijuana use Prevention and Cessation Program (10 Minutes)**

**1. Program Development:**

*We have used information from our focus groups in the past to design some programs for young people who want to quit vaping nicotine. We are trying to extend that program to potentially include marijuana vaping. We would want to know what feedback you might have for a program for youth/young adults who want to reduce or quit their marijuana vaping.*

What kind of things would be important for a program like this? What kind of things would make people want/not want to participate?

Possible probes:

- Confidentiality
- In person/online
- Group/individual setting
- Worry about what friends think (image issues)
- Location if in person
- Low motivation to quit
- Use role models to increase motivation (athletes, college students, teachers, family, community leaders, older teens, ex-vapers)
- Learn facts about the health effects
- Learn negative effects of *marijuana* versus gains from quitting
- Receive encouragement from family/friends to join
- Rewards for staying quit
- Time of day?
- Number of sessions/frequency of sessions
- Have too much responsibility outside of school
- How to motivate youth to quit

*How can* ***parents/school staff*** *be involved in a quit marijuana vaping program?*

Possible probes:

- Parents quit too
- Parents support teens in quitting
- Don’t involve parents at all
- Don’t involve staff
- Involve staff
- Concerns about staff/parents

**V. Ending Remarks (5 Minutes)**

*1. What have we missed?*

*2. What final thoughts or comments do you have?*

*Thank you all very much for your time and your input during this discussion. We value your opinions and hope that this information will assist us in understanding marijuana vaping among your age group.*
